# Update on the Mechanism and Treatment of Sevoflurane-Induced Postoperative Cognitive Dysfunction

**DOI:** 10.3389/fnagi.2021.702231

**Published:** 2021-07-08

**Authors:** Cong-mei Wang, Wei-can Chen, Yan Zhang, Shu Lin, He-fan He

**Affiliations:** ^1^Department of Anesthesiology, The Second Affiliated Hospital, Fujian Medical University, Quanzhou, China; ^2^Diabetes and Metabolism Division, Garvan Institute of Medical Research, Darlinghurst, Sydney, NSW, Australia; ^3^Centre of Neurological and Metabolic Research, The Second Affiliated Hospital of Fujian Medical University, Quanzhou, China

**Keywords:** combination therapy, neuroinflammation, neurotransmitter, postoperative cognitive dysfunction, sevoflurane anesthesia

## Abstract

Sevoflurane is one of the most widely used anesthetics for the induction and maintenance of general anesthesia in surgical patients. Sevoflurane treatment may increase the incidence of postoperative cognitive dysfunction (POCD), and patients with POCD exhibit lower cognitive abilities than before the operation. POCD affects the lives of patients and places an additional burden on patients and their families. Understanding the mechanism of sevoflurane-induced POCD may improve prevention and treatment of POCD. In this paper, we review the diagnosis of POCD, introduce animal models of POCD in clinical research, analyze the possible mechanisms of sevoflurane-induced POCD, and summarize advances in treatment for this condition.

## Introduction

Postoperative cognitive dysfunction (POCD) is a type of cognitive impairment that occurs after anesthesia and surgery. Patients with POCD experience impairment of cognitive abilities including attention, concentration, memory, information processing, executive function, visual-spatial ability, and psychomotor speed (Huang et al., 2020). POCD seriously affects the lives of patients, prolongs the length of hospitalization, increases medical expenses, and increases the burden on patients and their families (Cui et al., 2018). Among patients with apparently decent cognitive abilities before undergoing anesthesia and non-cardiac surgery, approximately 12% show symptoms of cognitive dysfunction after surgery (Needham et al., 2017).

Sevoflurane is one of the most frequently used volatile anesthetics and has the advantages of quick inhalation, rapid induction, and fine controllability. However, there is evidence that exposure of humans and animals to sevoflurane-based anesthetics, especially repeat exposure, can lead to neuropathological changes to the brain and long-term cognitive impairment (Tang et al., 2020). The neurotoxic effects of sevoflurane may be mediated via neuroinflammation, a neurotransmitter imbalance, and/or a reduction in brain-derived neurotrophic factor (BDNF) concentration (Gibert et al., 2012; Ozer et al., 2017; Cui et al., 2018; Qiu et al., 2018).

## Postoperative Cognitive Dysfunction

The potential association between POCD and surgery under general anesthesia was first described in 1955 (Bedford, 1955; Belrose and Noppens, 2019). Subsequent research on POCD has focused on different aspects of the condition, ranging from epidemiology to diagnosis and treatment, and the field continues to expand. POCD is common among the elderly and places an additional burden on patients’ family members and caregivers. The incidence of cognitive dysfunction ranges from 10 to 65% and varies with many factors such as age, education level, sex, comorbidities, type of surgery, and evaluation methods (Boone et al., 2020).

The definition and diagnostic criteria of POCD vary across time periods and disciplines. Therefore, consistent terminology is necessary for the identification and diagnosis of POCD. In 2018, a multiprofessional working group recommended use of the Diagnostic and Statistical Manual of Mental Disorders, fifth edition (DSM-5) nomenclature for clinical purposes; the recommended POCD index refers to a follow-up period from 30 days to 12 months after anesthesia and surgery, assuming that the cognitive decline cannot be explained by any other medical condition (Evered et al., 2018). Due to the variability of previous studies, in this review, the term POCD will be used interchangeably with postoperative delirium (POD)—a temporary state of cognitive change that occurs immediately or within a few days after surgery.

### Diagnosis

Diagnosis and research are prerequisites for treatment. Changes in behavioral and pathological biomarkers are the two major characteristics of POCD and the basis for its diagnosis. Although the 2018 recommendations provide a formal definition of POCD, there is a lack of uniform diagnostic criteria. A widespread criterion for POCD is a decline of one standard deviation from before to 3 months after surgery in at least two objectively measured cognitive functions, including verbal memory, attention, cognitive flexibility, language, and visuomotor abilities (Butz et al., 2019). The DSM-5 also specifies six key domains that should be considered when implementing diagnostic criteria for neurocognitive diseases: Perceptual-motor function, language, learning and memory, social cognition, complex attention, and executive function (Olotu, 2020). These functions are generally assessed using cognitive dysfunction scales in the postoperative period. The Mini–Mental State Examination, Montreal Cognitive Assessment, confusion assessment, and clock-drawing test are instruments commonly used to assess cognitive function in clinical settings. These assessment scales are applicable to all neurocognitive disorders, not just to POCD. Moreover, different scales focus on different cognitive domains. Therefore, it is difficult to determine which tests are most appropriate for diagnosing POCD. In practice, certain clinicians combine different scales for a holistic assessment, such as in the form of the Z score. The Z score is subsequently combined into a composite cognitive score by averaging the Z scores of each test from the patient’s preoperative assessments (Li Y. et al., 2019).

In addition to abnormal behavior, changes in the concentration of certain biomarkers may also indicate cognitive dysfunction. The biomarkers of POCD are primarily classified into brain-derived, inflammation-related, and neurotransmitter-based biomarkers. Tau protein, β-amyloid (Aβ), calcium binding protein β (S100β), and neuron-specific enolase (NSE) are specific markers of brain function. This may be why brain-derived biomarkers appear to be the gold standard for POCD. Further research has focused on inflammation-related biomarkers such as C-reactive protein, interleukins, and tumor necrosis factor-α (TNF-α). These markers are highly sensitive for the diagnosis and prediction of POCD but have low specificity. Neurotransmitters, metabolites, and their precursors are involved in cognition. However, the measurement of neurotransmitters after surgery cannot immediately distinguish patients with POCD from those without.

As postoperative cognition is a multifactorial process, there can be no single predictive marker, and clinicians typically examine several biomarkers simultaneously. A set of appropriately selected biomarkers with adequate sensitivity and specificity for POCD is needed (Schaefer et al., 2019; Majewski et al., 2020; Table 1).

**TABLE 1:** Reported biomarkers of postoperative cognitive dysfunction.

**Biomarkers**		**Localization**	**Specific**	**Relationship**	**References**
Brain-derived biomarkers	S100β	Astroglia, Schwann cells	✓	↑	Schaefer et al., 2019; Majewski et al., 2020
	NSE	Neuroendocrine cells, neural tissue	✓	↑	Schaefer et al., 2019
	Aβ	Axonal cytoskeleton	✓	↑	Majewski et al., 2020
	TAU	Axonal cytoskeleton	✓	↑	Schaefer et al., 2019
	BDNF	Cerebral blood vessels	✓	↓	Schaefer et al., 2019
Inflammation-related biomarkers	IL-1	Serum	✓	↑	Wang et al., 2018
	TNF-α	Serum	×	↑	Wang et al., 2014
	CRP	Serum	×	↑	Li Y. et al., 2019
	IL-6	Serum	×	↑	Wang et al., 2014
	IL-7	Serum	×	↑	Zhang X. et al., 2019
Neurotransmitter-based biomarkers	IGF-1	Serum	×	↓	Majewski et al., 2020
	AChE	Serum/cerebral blood vessels	✓	↑	Schaefer et al., 2019

**POCD, postoperative cognitive dysfunction; ✓*, *represents established markers specific for POCD; ×*, *represents unspecific markers; ↑*, *represents positive correlation with POCD; ↓*, *represents negative correlation with POCD; S100β, calcium-binding protein; NSE, neuron-specific enolase; Aβ, β-amyloid; Tau, a microtubule-stabilizing protein; BDNF, brain-derived neurotrophic factor; IL, interleukin; TNF-α, tumor necrosis factor-α; CRP, C-reactive protein; IGF-1, insulin growth factor-1; AChE, acetylcholinesterase.**

### Animal Models of POCD

A POCD animal model was established based on the different mechanisms of POCD caused by surgery or anesthetics. POCD in an animal model is mainly measured by analyzing animal behavior. The Morris water maze, open field test, Y-maze training and test, and novel object recognition task, among others, are common behavior-testing methods. Pathological examination of animal brain tissues and the evaluation of various *in vivo* markers are objective methods to further verify whether establishment of a model is successful.

The surgical POCD model is based on the fact that acute inflammatory reactions occur after all surgeries. Inflammation-induced cognitive impairment has been recognized for decades. Surgical procedures to induce POCD include orthopedic procedures such as internal fixation of tibial fractures (Miller-Rhodes et al., 2019; Yang et al., 2019), exploratory laparotomy (Zhang Q. et al., 2018; Zhang Z. et al., 2018), bilateral carotid artery ligation (Yamamoto et al., 2018; Zhang et al., 2021), splenectomy (Kamer et al., 2012), partial hepatectomy (Wei et al., 2018), and cardiac surgery (Hovens et al., 2016). Although different types of surgery can induce POCD in rats, the specific aspects and degrees of cognitive effects are not consistent among them. Some scholars have found that both abdominal and cardiac surgery can impair spatial memory, but only cardiac surgery can impair spatial learning and object recognition. Moreover, the effects of abdominal surgery appear to be limited to the hippocampus, whereas cardiac surgery appears to be associated with more widespread changes in the brain (Hovens et al., 2016). The clinically relevant tibial fracture mouse model is the most common POCD model, and this may be related to the high incidence of POCD in clinical orthopedic surgery. Indeed, as many as 50% of elderly patients suffer neurological complications after routine orthopedic surgery for fracture repair (Xiong et al., 2018).

N-Methyl-D-aspartic acid (NMDA)- and γ-aminobutyric acid (GABA)-mediated pathways play a critical role in normal neurodevelopment (Mutch et al., 2018). These two pathways are the targets for most anesthetics, and animal models of POCD can be established by performing anesthetic treatment. The primary anesthetic drugs used in the study of animal models of POCD are inhaled anesthetic gases such as sevoflurane (Huang C. et al., 2018) and isoflurane (Zhu et al., 2018; Velagapudi et al., 2019), intravenous anesthetics such as ketamine (Kozela et al., 2020) and propofol (Li X. et al., 2019), and adjuvant drugs such as scopolamine (Qiu et al., 2016). Anesthetic-induced cognitive impairment may depend on the developmental stage, anesthetic agent, and exposure dose. Researchers have reported that repeated but no single exposure to sevoflurane can cause POCD (Shen et al., 2013). The incidence of sevoflurane-induced POCD in adult mice is lower than that in young mice (Shen et al., 2013). Compared with normal, saline-administered, and low-dose scopolamine-administered rats, rats infused with 1.8 mg/kg scopolamine required more time to complete behavioral activity tests (Qiu et al., 2016; Table 2).

**TABLE 2:** Animal models of postoperative cognitive dysfunction.

**Animals**	**Experimental models**	**Cognitive testing**	**References**
32-month-old rats	Sevoflurane	Novel object recognition test Y-maze test	Qin et al., 2018
18-month-old rats	Isoflurane	Morris water maze test Open field test	Zhu et al., 2018
17–18-month-old rats	Propofol	Novel object recognition test Contextual fear—conditioning test	Kozela et al., 2020
10–14-week-old mice	Isoflurane Internal fixation of tibial fractures	Contextual fear—conditioning test	Yang et al., 2019
12–14-month-old mice	Isoflurane Exploratory laparotomy	Open field test Contextual fear—conditioning test	Zhang Z. et al., 2018
6-week-old rats	Isoflurane Bilateral carotid ligation	Morris water maze test	Zhang et al., 2020
6–8-week-old mice	Isoflurane Splenectomy	Novel object recognition test	Kamer et al., 2012

Although POCD animal models can be established independently by using anesthesia or surgery, researchers tend to combine these two methods. Two interesting phenomena occur in POCD animal models. First, the evidence for anesthetic neurotoxicity is unequivocal when studied in animal models. However, these findings have translated poorly to the clinical domain when equated to POD in adults and POCD in either children or the elderly (Mutch et al., 2018). Therefore, it is necessary to control for age and pre-existing diseases when constructing POCD animal models. Second, POCD models are currently limited to rats and mice. No studies on POCD in non-human primates have been reported (Eckenhoff et al., 2020).

## Sevoflurane-Inhalation Anesthesia

Sevoflurane (chemical name: 1, 1, 1, 3, 3, 3,-hexafluoro-2-[fluoromethoxy]propane ether) is a colorless, fragrant liquid first synthesized by Regan in 1968 (Smith et al., 1996). This powerful inhalation anesthetic is widely used in clinics, especially for children. However, its anesthetic mechanism remains unclear. Sevoflurane reportedly causes amnesia, analgesia, coma, and quiescence, primarily by inhibiting NMDA receptors (Petrenko et al., 2014). In addition, *in vivo* studies have suggested that GABA type A (GABAA) receptors (Lim et al., 2014), nicotinic acetylcholine receptors (Tang et al., 2018), and voltage-gated sodium channels (Yokoyama et al., 2011) are potential targets for sevoflurane related to its hypnotic effects (Palanca et al., 2017).

The clinical application of sevoflurane is complicated. It is currently the most commonly used inhalation anesthetic in operating rooms. It yields excellent respiratory tolerance and hemodynamic stability, providing a safe anesthetic process. However, electroencephalographic signs of epilepsy have been observed with sevoflurane, both during induction (Julliac et al., 2013) and under steady-state conditions (Cui et al., 2018). Sevoflurane treatment statistically significantly increases the incidence of POCD, particularly in the elderly.

Due to improvements in the overall standard of living, medical care, nutrition, and education, older patients now account for an increasing proportion of the surgical population. In one study, the incidence of POCD was at least twice as high in individuals older than 60 years as in younger age groups (Alalawi and Yasmeen, 2018). The high incidence of POCD in older individuals may be related to specific susceptibility factors. First, aging itself is a risk factor for cardiovascular, respiratory, renal, and neurodegenerative diseases. Immune responses to pathological insults also decrease with age. Second, the pharmacokinetics and pharmacodynamics of older individuals are considerably altered compared to younger individuals. With gradual degeneration of various organ functions, sevoflurane-based anesthesia in older individuals results in a lower minimum alveolar concentration of sevoflurane and an increased cumulative effect of sevoflurane (Cooter et al., 2020). Hence, sevoflurane may remain in the blood for longer periods in older than in younger individuals after anesthesia. The fragile balance between neuroinflammation and neuronal functioning in older individuals is easily interrupted upon pathological insult (Luo et al., 2019). Studies have found elevated plasma concentrations of S-100β protein, TNF-α, and IL-6 in individuals receiving sevoflurane anesthesia (Qiao et al., 2015). Sevoflurane has been found to induce increased inflammation and apoptosis of hippocampal neurons in older rats (Yang L. H. et al., 2020). Sevoflurane-induced anesthesia upsets the balance between neuroinflammation and neuronal functioning, especially in older individuals, increasing the incidence of POCD. The appropriate management of pre-existing diseases and maintenance of optimum bodily functioning may be an effective way to reduce the incidence of sevoflurane-induced POCD. Finally, in older animals, postanaesthetic sevoflurane-related behavioral deficits tend to be larger and last longer than those in younger animals (Peng et al., 2020).Therefore, it is important that studies include aged rodents when performed to investigate the mechanism of sevoflurane-induced POCD.

In addition to patient-related factors, the type of general anesthetic used for maintenance of anesthesia may affect the incidence of POCD. Sevoflurane-induced anesthesia may predispose patients to POCD. Qiao et al. (2015) discovered that the incidence of POCD was higher in older patients who underwent resection of esophageal carcinoma under inhalational anesthesia with sevoflurane than in those in whom anesthesia was maintained with intravenous propofol. In another randomized controlled trial of laparoscopic cholecystectomy, sevoflurane-induced anesthesia aggravated POCD compared to propofol (Geng et al., 2017). Tachibana et al. (2015) investigated the quality of cognitive function in older patients undergoing an extended period of desflurane or sevoflurane anesthesia. They discovered the Mini-Mental State Examination score, which represents learning by repeat testing, was reduced following sevoflurane but not desflurane anesthesia.

The contradictory effects of sevoflurane have prompted researchers to study its effects on neurons. Some studies have confirmed that sevoflurane exerts neuroprotective effects through specific pathways. In a rat model of focal cerebral ischemia, sevoflurane pre-treatment exerted a neuroprotective effect by reducing Akt signaling activity and activating autophagy (Lu G. et al., 2020). Kim et al. (2017) demonstrated that post-treatment with sevoflurane reduces apoptosis by activating phosphorylation of the Janus kinase 2-signal transducer and activator of transcription 3 pathway, upregulating Bcl-2 (an anti-apoptotic protein) and downregulating Bax (a pro-apoptotic protein) (Kim et al., 2017). In addition, sevoflurane post-treatment can upregulate mir-203 expression to attenuate cerebral ischemia–reperfusion-induced neuroinflammation by targeting *MyD88* (Zhong H. et al., 2020). However, more studies have been conducted on sevoflurane-induced neuropathy and its effect on postoperative cognitive function, the details of which are discussed in the following section.

## Sevoflurane-Inhalation Anesthesia and POCD

POCD is a multifactorial, neurodegenerative condition of which the underlying mechanisms remain unclear. A series of animal studies and repeated clinical trials have shown that after exposure to sevoflurane-inhalation anesthesia, humans and animals experience varying degrees of cognitive decline. There are many hypotheses regarding the pathogenesis of sevoflurane-induced POCD, including neuroinflammation, changes in neurotransmitters, a decrease in BDNF, mitochondrial oxidative stress, and changes in Aβ concentrations. These mechanisms are not completely independent and interact with each other. In recent years, increasing attention has been paid to the relationship between sevoflurane-induced POCD and neuroinflammation, changes in neurotransmitters, and BDNF reduction.

### Neuroinflammation-Related Sevoflurane-Induced POCD

Neuroinflammation in the brain, particularly in the hippocampus, has been shown to play a contributory role in POCD. The activation of microglia may play a key role in the occurrence of POCD as activated microglia are now recognized as the main source of pro-inflammatory cytokines and chemokines in the central nervous system (CNS) (Block et al., 2007; Lu B. et al., 2020). Neuroinflammation and microglial activation trigger and amplify a complex cascade of reactions, including immune response activation, microcirculatory changes, increased hippocampal oxidative stress, and increased blood–brain barrier permeability (Su et al., 2020). The pro-inflammatory cytokines IL-1β, IL-6, and TNF-α were statistically significantly increased in the brains of rats exposed to sevoflurane in a number of studies (Wadhwa et al., 2017; Huang L. et al., 2018). The mechanisms by which sevoflurane induces neuroinflammation are worth exploring.

Nuclear factor-kappa B (NF-κB) is a family of dimeric transcription factors that recognize and regulate genes involved in inflammation. NF-κB is normally retained in the cytoplasm by binding to NF-κB inhibitors (IκB). Various receptor-mediated signaling cascades activate the IκB kinase complex, which subsequently phosphorylates the inhibitory cytoplasmic NF-κB chaperone IκB, thus allowing NF-κB dimers to translocate to the nucleus and initiate specific gene transcription (Wang et al., 2014; Afonina et al., 2017; Huang L. et al., 2018). Sevoflurane is highly lipophilic and easily diffuses across the cell membrane without binding to specific receptors on the cell membrane, which may directly stimulate NF-κB signaling. Sevoflurane has been shown to increase intracellular Ca^2+^ by activating GABA receptors, inducing mitochondrial damage, and increasing the levels of intracellular reactive oxygen species (ROS) (Zhu et al., 2021). A sevoflurane-induced increase in intracellular Ca^2+^ may activate NF-κB signaling and lead to increased concentrations of pro-inflammatory cytokines (Shen et al., 2013). IL-17A, a novel cytokine, increases statistically significantly in the hippocampus of sevoflurane-induced aged rats and can promote the binding of Act1 (an activator of NF-κB) and IL-17R to induce activation of the NF-κB signaling pathway (Yang and Yuan, 2018).

**FIGURE 1:**
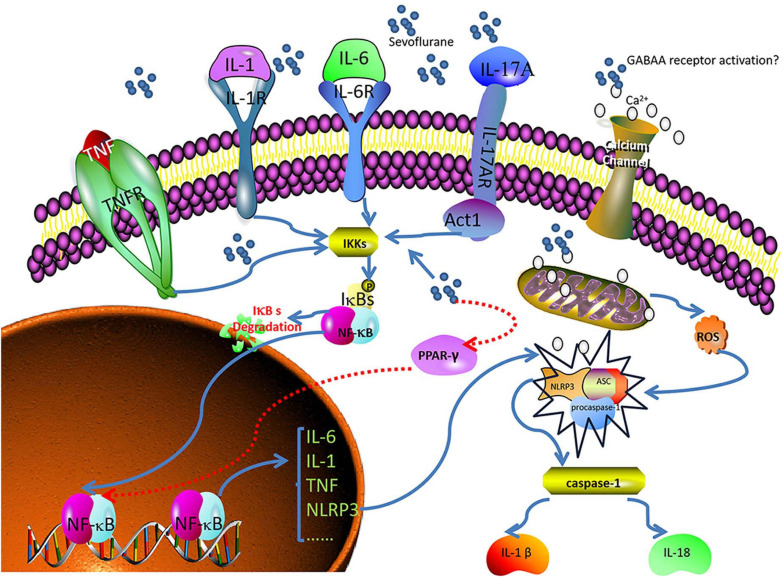
Sevoflurane induces neuroinflammation and leads to POCD. Nuclear factor-kappa B (NF-κB) recognizes and regulates genes related to inflammation. Peroxisome proliferator-activated receptor- γ (PPAR-γ) exerts anti-inflammatory effects by inhibiting NF-κB. The Nod-like receptor protein 3 (NLRP3) inflammasome regulates the maturation and release of pro-inflammatory cytokines. Sevoflurane directly penetrates the cell membrane and stimulates NF-κB signaling. Sevoflurane downregulates PPAR-γ, increases intracellular calcium levels, induces mitochondrial injury, and increases reactive oxygen species (ROS). Calcium and ROS, in turn, activate NLRP3, and interleukin (IL)-1, IL-6, tumor necrosis factor (TNF), and IL-17 activate NF-κB.

Peroxisome proliferator-activated receptor-γ (PPAR-γ) is a ligand-inducible transcription factor of the nuclear hormone receptor family and is expressed in several cell types in the brain, including microglia, astrocytes, and neurons. PPAR-γ activation exerts anti-inflammatory effects by inhibiting NF-κB (Zhang X. et al., 2019). Dong et al. (2018) confirmed that sevoflurane could aggravate neuroinflammation mediated by microglia by downregulating hippocampal PPAR-γ, thereby exacerbating cognitive dysfunction (Lv et al., 2017; Dong et al., 2018). The Nod-like receptor protein 3 (NLRP3) inflammasome is essential to the immune response, and includes NLRP3, apoptosis-associated speck-like protein containing a caspase-recruitment domain, and procaspase-1. It regulates the maturation and release of pro-inflammatory cytokines IL-1 and IL-18 by cleaving caspase-1, leading to the secretion of many inflammatory factors (Shao et al., 2020). NLRP3 inflammasome activation requires two steps. First, the NF-κB pathway upregulates NLRP3 transcription. Second, the NLRP3 inflammasome is assembled and activated (Wang et al., 2018). Mitochondrial damage and subsequent release or exposure to mitochondrial contents—such as ROS—are essential for the assembly of the NLRP3 inflammasome (Wei et al., 2019; Ye et al., 2019). Calcium influx is also thought to be required for optimal activation of the NLRP3 inflammasome (Afonina et al., 2017). In the sevoflurane model, calcium influx and increased exposure to ROS can lead to the activation of the NLRP3 inflammasome, which may influence the neurological outcome (Ye et al., 2019; Figure 1).

Sevoflurane can induce neuroinflammation through multiple pathways. The causes of POCD are complex, and neuroinflammation may be an important bridge connecting sevoflurane and POCD.

### Brain-Derived Neurotrophic Factor-Related Sevoflurane-Induced POCD

Neurotrophins are also critical in the development of POCD. BDNF is a widely studied neurotrophic factor mediated by tyrosine kinase B (TrkB). It plays a key role in neuronal survival, plasticity, neurogenesis, and synapse formation in the developing brain (Wei et al., 2018). Converging evidence strongly suggests that deficits in BDNF signaling contribute to the pathogenesis of several major diseases and disorders such as Huntington’s disease, Alzheimer’s disease, and depression (Lu et al., 2014). An association between BDNF and POCD has also been reported. Patients or experimental animals with lower concentrations of BDNF exhibit more pronounced symptoms of POCD. Therefore, BDNF has become an important biomarker of POCD (Tian X.S. et al., 2015; Hem et al., 2016; Wu et al., 2016).

The relationship between sevoflurane and BDNF has been a topic of discussion in recent years. Many researchers believe that sevoflurane has a direct or indirect effect on BDNF expression. Xu et al. (2020) reported that sevoflurane treatment resulted in inhibition of BDNF expression. Tang et al. (2020) found that exposure of the hippocampus of developing mice to sevoflurane inhibited the protein sirtuin 1 (SIRT1), which was associated with the downregulation of BDNF.

Researchers have provided evidence that abnormal regulation of the BDNF/TrkB signaling pathway is mediated by NMDA receptor/Ca^2+^/calpain in the occurrence of POCD in aging mice (Qiu et al., 2020). Many previous studies have confirmed that NMDA receptors may be targeted by sevoflurane (Zhang et al., 2016) and that sevoflurane can increase the intracellular Ca^2+^ concentration (Qiu et al., 2020). Therefore, it is reasonable to believe that sevoflurane induces POCD by regulating BDNF/TrkB signaling through the NMDA receptor/Ca^2+^/calpain pathway. Hence, upregulation of the expression of BDNF is an attractive method to pursue in treating sevoflurane-induced POCD in the future.

### Neurotransmitter-Related Sevoflurane-Induced POCD

Cognitively impaired rats show increased levels of serum pro-inflammatory factors and changes in the concentrations of prefrontal cortex and hippocampal neurotransmitters such as dopamine, epinephrine, serotonin, acetylcholine (ACh), and GABA (Ding et al., 2021). α-Synuclein, a protein containing 140 amino acids, is primarily located at the presynaptic terminal and maintains neurotransmitter homeostasis. Anesthesia and surgery can inhibit hippocampal cell autophagy, increase α-synuclein oligomerization, cause a neurotransmitter imbalance, and induce POCD (Yang N. et al., 2020). Since sevoflurane regulates the CNS and induces anesthesia by targeting different neurotransmitters and receptors on the synapses (Qin et al., 2018; Yin et al., 2019), its effect on various neurotransmitters is another cause of sevoflurane-induced POCD.

#### Acetylcholine

Acetylcholine (ACh) is a major excitatory neurotransmitter involved in cognitive processes (Coppi et al., 2021). Preoperative use of anticholinergic medications (such as atropine and scopolamine) is associated with an increased risk of POCD (Qiu et al., 2016). Acetylcholinesterase (AChE) is an enzyme that decomposes ACh into acetate and choline, and is located in synaptic clefts in the brain. The use of AChE inhibitors elevates the concentration of Ach in treated tissues and can enhance cognitive ability (Chen et al., 2018). Moreover, the alpha 7 nicotinic acetylcholine receptor (α7nAChR) plays an important role in regulating the balance between the pro- and anti-inflammatory states in the body (Yin et al., 2019). Many therapies enhance postoperative cognitive ability by upregulating Ach receptors and activating the α7nAChR-mediated cholinergic anti-inflammatory pathway (Liu et al., 2017; Wang T. et al., 2019).

The release of neurotransmitters is determined by the amount of Ca^2+^ entering the nerve terminal, which is related to Na^+^, Ca^2+^, and K^+^ channels (Westphalen et al., 2013). Sevoflurane has a wide range of effects on voltage-dependent ion channels (Yokoyama et al., 2011; Liu Y. et al., 2016; Fukushima et al., 2020). Chen et al. (2015) suggested that exposure to sevoflurane could reduce the transmission of cholinergic synapses by inhibiting the Ca^2+^ current. Furthermore, Silva et al. (2005) proposed that sevoflurane could promote the release of ACh in the cerebral cortex of rats by releasing Ca^2+^. Sevoflurane has also been shown to decrease the expression of the ACh receptor in the hippocampus, although it was unclear whether that pathway affected Na^+^ channels (Peng et al., 2012). In brief, sevoflurane-induced POCD may be considered from the perspective of ACh in the following manner: Sevoflurane inhibits the release of ACh and reduces its neuroexcitatory effect; it also downregulates Ach receptors and inhibits their anti-inflammatory effect.

#### γ-Aminobutyric Acid

GABA is the chief inhibitory neurotransmitter in the human CNS (Maldonado, 2013), and the GABAergic system controls the excitability of neuronal networks. The GABAA receptors are the main targets of sevoflurane; such binding is related to the development of cognitive memory deficits after anesthesia (Zurek et al., 2012; Li T. et al., 2019). After mice undergo inhalation anesthesia, their concentration of GABA decreases and their level of surface GABAA receptor proteins increases (Zhang et al., 2020). The Na–K–Cl–1 cotransporter (NKCC1) is a chloride importer that maintains high intracellular chloride levels, establishing a concentration gradient across neuronal membranes that drives a chloride efflux upon GABAA receptor activation. The net efflux of chloride depolarizes neurons, during which GABA acts as an excitatory neurotransmitter. In other words, NKCC1 excites GABA. This effect can be reversed by the chloride extruder, K–Cl–2 cotransporter (KCC2), which causes GABA to revert to an inhibitory state. Exposure to sevoflurane can upregulate NKCC1 and downregulate KCC2, thus affecting the excitability of GABA in the neonatal mouse brain (Cabrera et al., 2020). The expression levels of miR-30a, miR-31, miR-190a, and miR-190b were statistically significantly decreased in the hippocampus of aged rats exposed to sevoflurane, whereas the protein concentrations of GABAA receptors were statistically significantly increased. Thus, the miRNA-GABAergic transmission pathway may be involved in the pathophysiological alterations characterized by sevoflurane-induced POCD (Shan et al., 2017; Xu et al., 2020).

#### Dopamine

Dopamine is a monoamine neurotransmitter that plays a key role in cognition and movement. There are five subtypes of dopamine receptors (D1–D5), of which D1 and D2 receptors are most abundantly expressed in the brain and appear to be mainly involved in arousal (Kelz et al., 2019). Previous studies have shown that activation of dopamine D1 receptors can induce emergence from general anesthesia (Taylor et al., 2013). Dopamine D2 receptor antagonists deepen sevoflurane anesthesia. Dopamine acts on the D2 heteroreceptors of GABAergic neurons, causing hyperpolarization, which leads to increased activity in the neural network by reducing inhibitory GABA activity (Araki et al., 2018).

Sevoflurane anesthesia enhances the effect of the psychotropic agent-induced acceleration of dopamine turnover in the brain (Taharabaru et al., 2018). Furthermore, the expression levels of the dopamine receptor genes are increased by exposure to sevoflurane (Hayase et al., 2016).

#### Hydroxytryptamine

Hydroxytryptamine (5-HT), also known as serotonin, is distributed in various areas of the brain and has long been associated with various behavioral functions, especially mood regulation, aggression, and impulsivity. Experimental studies on animals and humans have revealed that 5-HT may play an important role in normal and disturbed cognitive function. The physiological role of 5-HT_3_ receptors in controlling the release of neurotransmitters such as dopamine, ACh, GABA, or 5-HT itself may be an important factor affecting cognition (Faerber et al., 2007).

Among the many recognized 5-HT receptors, 5-HT_1A_ and 5- HT_3_ receptors are targets for sevoflurane, the binding of which enhances their activation (Suzuki et al., 2002; Hang et al., 2010; Qiu et al., 2018). Surgical patients, especially children, receiving sevoflurane frequently experience restlessness in the postoperative period (Kanaya et al., 2014). 5-HT_1A_ receptor agonists can reduce numerous aspects of aggressiveness in mice and rats (Bacqué-Cazenave et al., 2020). Neuropeptide Y (NPY), acting through Y1 receptors, regulates the 5-HT system and coordinates aggressive behavior (Karl et al., 2004). Moreover, hippocampal NPY concentrations decrease after sevoflurane treatment (Kang et al., 2020). These results suggest that 5-HT and NPY may be involved in sevoflurane-induced postoperative agitation. Sevoflurane can also alter the expression of 5-HT_1A_ receptors. 5-HT_1A_ receptors regulate the expression of the second messenger cyclic adenosine monophosphate (cAMP) by activating adenylate cyclase; cAMP, in turn, activates protein kinase A (PKA). Furthermore, activated PKA phosphorylates the corresponding S133 site of the cAMP-responsive element-binding protein, which is involved in regulating learning and memory (Qiu et al., 2018), activating its transcriptional activity.

Sevoflurane non-competitively inhibits 5-HT_3_ receptors (Suzuki et al., 2002) and modulates currents mediated by them (Stevens et al., 2005). Sevoflurane sequesters acrolein, a lipid peroxidation product, associated with aging, that is elevated in the brains of elderly people. The enhanced partitioning of acrolein increases its focal concentration (and hence its reactivity to serotonin), enhancing the formation of a serotonin-derived melanoid (SDM). SDM exhibits redox activity, which can destroy the lipid bilayer and cause neuronal damage (Roberts et al., 2007; Miller et al., 2010; Brownrigg et al., 2011; Table 3).

**TABLE 3:** Possible mechanisms of sevoflurane-induced postoperative cognitive dysfunction.

**Kernels**	**Possible mechanisms**	**References**
Ca^2+^, NF-κB	Sevoflurane activates the NF-κB signaling pathway by increasing intracellular Ca^2+^ and IL-17A content, elevating the concentrations of proinflammatory cytokines	Shen et al., 2013
IL-17A, NF-κB		Yang and Yuan, 2018
PPAR-γ, NF-κB	Sevoflurane activates the NF-κB signaling pathway by downregulating PPAR-γ, elevating the concentrations of proinflammatory cytokines	Dong et al., 2018; Zhang X.P. et al., 2019
ROS, Ca^2+^, NLRP3	Sevoflurane activates the NLRP3 signaling pathway by increasing intracellular Ca^2+^ and ROS content, elevating the concentrations of proinflammatory cytokines	Ye et al., 2019
SIRT1, BDNF	Sevoflurane downregulates BDNF by inhibiting the expression of SIRT1, which in turn affects neuronal survival, neuronal plasticity, neurogenesis, and synapse formation.	Tang et al., 2020; Xu and Qian, 2020
AChE, ACh	Sevoflurane upregulates the expression of AChE, decreases the concentration of Ach, and downregulates the cholinergic anti-inflammatory pathway.	Yin et al., 2019
GABA, GABAA receptors	Sevoflurane decreases the concentration of GABA and potentiates GABAA receptors	Cabrera et al., 2020; Zhang et al., 2020
Dopamine, dopamine receptors	Sevoflurane accelerates dopamine turnover in the brain and increases the expression levels of the dopamine receptor genes	Hayase et al., 2016; Taharabaru et al., 2018
Serotonin, SDM	Sevoflurane sequesters acrolein and may promote the production of SDM that depletes local serotonin and enhances neuronal vulnerability	Roberts et al., 2007; Miller et al., 2010; Brownrigg et al., 2011

**NF-κB, nuclear factor-kappa B; IL, interleukin; PPAR-γ, peroxisome proliferator-activated receptor-γ; ROS, reactive oxygen species; NLRP3, Nod-like receptor protein 3; SIRT1, sirtuin 1; BDNF, brain-derived neurotrophic factor; AChE, acetylcholinesterase; Ach, acetylcholine; GABA, γ-aminobutyric acid; GABAA, GABA type A; SDM, serotonin-derived melanoid.**

## Advances in Treatment

At present, the treatment of POCD mainly relies on drug therapy and rehabilitation therapy such as acupuncture. These treatments are focused on reducing inflammatory responses, maintaining the balance of neurotransmitters, regulating receptor excitability, and increasing the BDNF concentration.

### Narcotic-Related Drugs

The effectiveness of narcotic-related drugs as treatment for POCD is controversial. Dexmedetomidine (Dex) is gaining increasing recognition for the prevention and alleviation of POCD. It is a highly selective α_2_-adrenergic receptor agonist used for short-term sedation and analgesia in certain preoperative settings (Shi et al., 2020). Dex exerts anti-inflammatory effects and can effectively reduce POCD in humans and rats (Carr et al., 2018; Chen et al., 2019; Zhang D.F. et al., 2019). Studies have shown that Dex can stabilize the integrity of the blood-brain barrier and reduce apoptosis. These protective effects may be mediated by reducing the activation of the NF-κB and NLRP3 inflammasome pathways (Zhang X.P. et al., 2019). In addition, Dex enhances the cholinergic anti-inflammatory pathway via α7nAChR (Carr et al., 2018). The neuroprotective effect of Dex may be achieved by upregulating BDNF expression (Lv et al., 2016).

Etomidate, a hypnotic drug, is used by many doctors for rapid induction of anesthesia for intubation. It has been confirmed that etomidate mitigates NF-κB activation and pro-inflammatory cytokine production in rat macrophages (Liu M. et al., 2016). Compared with treatment with dexamethasone etomidate, Dex combined with etomidate yields a more satisfactory therapeutic effect in the treatment of older rats with POCD, effectively improving cognitive dysfunction and alleviating stress-related inflammation (Yu and Xie, 2020).

The κ-opioid receptor agonist, oxycodone, an opioid widely used for postoperative pain, can downregulate the expression of inflammatory factors and attenuate POCD (Gan et al., 2020; Fan et al., 2021).

Ketamine, a sedative with an analgesic effect, is widely used in orthopedic surgery in patients who concurrently sustained burn injuries. The brain-derived biomarkers, S100β and NSE, decrease after ketamine administration (Hollinger et al., 2021). The neuroprotective effect of ketamine may also be related to a reduction in postoperative systemic inflammation. Ketamine can also inhibit the expression of NF-κB, which is involved in the transcription of genes encoding pro-inflammatory cytokines (Hudetz et al., 2009).

### Anti-inflammatory Drugs

The reduction of inflammation is a direct method of improving POCD. Minocycline is commonly used to treat infectious diseases in the clinic owing to its anti-inflammatory effects. Attenuation of neuroinflammation may be mediated through the NF-κB signaling pathway, thereby improving POCD (Tian Y. et al., 2015; Wadhwa et al., 2017). Cyclooxygenase-2 (COX-2) is an enzyme mainly involved in inflammation and is induced by TNF-α and IL-1. Parecoxib (Wang Y.B. et al., 2019; Huang et al., 2020) and meloxicam (Kamer et al., 2012; Haile et al., 2016) are selective COX-2 inhibitors that reduce the incidence of POCD, which may be attributed to their anti-inflammatory effect.

### Antipsychotic Drugs

Antipsychotic drugs have been reported to attenuate POCD. Both galantamine and huperzine A are AChE inhibitors that reverse cognitive dysfunction by reducing the protein levels of IL-1β and normalizing excitatory synaptic transmission; huperzine A also increases the BDNF concentration (Mao et al., 2016; Wang T. et al., 2019; Cai et al., 2020). Haloperidol is a dopamine receptor antagonist that reduces excessive dopaminergic activity during wakefulness, thereby reducing e.g., aggressive behavior (Schrader et al., 2008; Nishigaki et al., 2019). Amantadine (Zhong J. et al., 2020) and amitriptyline (Hu et al., 2010) attenuate POCD by upregulating BDNF in the hippocampus.

### Acupuncture

As an integral part of traditional Chinese medicine, acupuncture has been practiced widely in China for thousands of years and is recommended by the World Health Organization as an alternative and complementary strategy for treatment (Yang F. M. et al., 2020). Acupuncture has been suggested as an effective intervention for many neurological disorders (Lu et al., 2016; Du et al., 2018). Since acupuncture is non-pharmacologically based, it carries with it no concerns regarding dependence, addiction, tolerance, and neurological toxicity, and its use does not increase the metabolic burden on the liver and kidneys. The application of electroacupuncture (EA) and acupuncture in the prevention and treatment of POCD has received increasing attention (Ho et al., 2020; Ye et al., 2021). Previous studies have shown that acupuncture can reduce markers of inflammation and nerve damage, providing solid evidence that it may protect the brain. Research by Liu et al. (2017) has shown that EA relieves cognitive dysfunction by increasing the expression of α7nAChR and activating the cholinergic anti-inflammatory pathway (Wang et al., 2012; Liu et al., 2017). The inhibition of the NF-κB signaling pathway in microglia and the resulting reduction of inflammation is another mechanism by which EA alleviates POCD (Han et al., 2015). Acupuncture has been found to play an important role in reducing the production of ROS (Jung et al., 2016) and COX-2 (Gondim et al., 2012). Recent data on the correlation between neurotrophins and acupuncture have shown that EA may relieve certain neuropathological disorders by modulating BDNF and its signaling pathway (Lin et al., 2014).

### Others

In addition to the aforementioned treatments, researchers are actively seeking new and effective treatments for POCD. Traditional Chinese medicine is a Chinese cultural heritage, and its effect on POCD has attracted attention. Resveratrol is a natural herb that is often used as an activator of SIRT1. SIRT1 is abundantly expressed in the hippocampal neurons and maintains mitochondrial function, alleviates inflammation (Zhao et al., 2020), and increases BDNF concentrations (Tang et al., 2020). Plants belonging to the *Cistanche* genus (*Cistanche* spp.; “Rou Cong Rong” in Chinese) have been used to prepare tonics in China for many years. *Cistanches*. can regulate PPAR-γ-dependent antioxidative and anti-inflammatory effects in rats (Peng et al., 2020). Both resveratrol and *Cistanches* have been proven to reduce sevoflurane neurotoxicity in rats. Therefore, Chinese herbal medicine may be effective in relieving sevoflurane-induced POCD.

NPY, a neuroprotective peptide, may be a potential target for POCD therapy. The hippocampus is very sensitive to insults and is commonly involved in cognitive impairment (Fang et al., 2016). NPY and its receptors are highly expressed in the hippocampus and NPY is released as a co-transmitter with neurotransmitters such as GABA (Méndez-Couz et al., 2021). Moreover, NPY exerts anti-inflammatory effects via Y1/Y2 receptors (Wheway et al., 2007; Wang W. et al., 2019).

**FIGURE 2:**
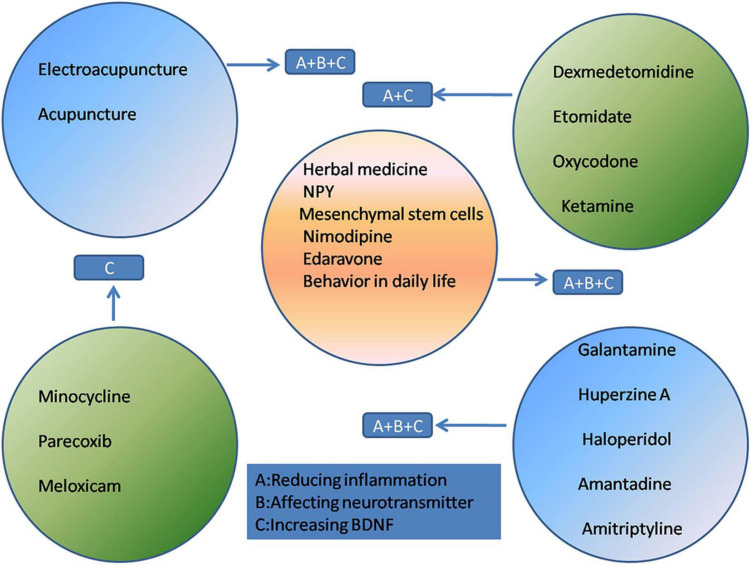
The treatment and mechanism of POCD.

Nimodipine, a calcium channel blocker, reverses sevoflurane toxicity and relieves POCD (Cui et al., 2018). Mesenchymal stem cell-conditioned medium (MSC-CM) ameliorates POCD in mice. The therapeutic effects of MSC-CM in a mouse model of POCD were associated with a reduction in inflammation, attenuation of oxidative stress, and an increased in BDNF expression in brain tissues (Jiang et al., 2019). Edaravone, a powerful free radical scavenger, can attenuate POCD in aged mice. The cognitive enhancement effect of edaravone may be due to its inhibition of neuroinflammation and increase of synaptic protein concentration and cholinergic transmission (Zhou et al., 2020). Even changes in behavior in daily life, such as caloric restriction, can improve cognitive function by promoting the expression of SIRT1 (Zhao et al., 2020; Figure 2).

## Summary and Future Prospects

Sevoflurane has become the most commonly used anesthetic because of its unique pharmacological properties. However, there is increasing evidence of a causal relationship between sevoflurane and POCD. Sevoflurane-induced POCD may be associated with neuroinflammation, a neurotransmitter imbalance, and a decreased BDNF concentration. Therefore, inhibiting inflammatory pathways, activating anti-inflammatory pathways, increasing the BDNF concentration, and maintaining a balance in neurotransmitters are aspects on which to focus in the treatment of sevoflurane-induced POCD.

Sevoflurane-based induction of POCD is a complex pathological process. At present, although there are many reports on the mechanism and treatment of sevoflurane-induced POCD, no cure has been found. Therefore, future research should be aimed at further clarifying the pathogenesis of sevoflurane-induced POCD and developing an effective combination therapy.

## Author Contributions

C-MW, H-FH, and SL contributed to the conception and design of the review. C-MW wrote the first and final draft of the manuscript. H-FH and SL contributed equally to the writing of the review and revised the manuscript and provided critical advice on the content. All authors contributed to the article and approved the final version.

## Conflict of Interest

The authors declare that the research was conducted in the absence of any commercial or financial relationships that could be construed as a potential conflict of interest.
